# 5-alpha reductase inhibitors (5-ARi) with or without alpha-blockers (α-B) for Benign Prostatic Hyperplasia do NOT lower the risk of incident Bladder Cancer: United States insurance claims data

**DOI:** 10.1007/s00345-023-04551-4

**Published:** 2023-08-07

**Authors:** Francesco Del Giudice, Federico Belladelli, Frank Glover, Satvir Basran, Shufeng Li, Evan Mulloy, Benjamin Pradere, Francesco Soria, Wojciech Krajewski, Rajesh Nair, Wade Muncey, Nicolas Seranio, Michael L. Eisenberg

**Affiliations:** 1 Department of Maternal Infant and Urologic Sciences, “Sapienza” University of Rome, Policlinico Umberto I Hospital, Rome, Italy; 2 Department of Urology, Stanford University School of Medicine, Stanford, CA USA; 3 University Vita-Salute San Raffaele, Milan, Italy; 4 Division of Experimental Oncology/Unit of Urology, Urological Research Institute, IRCCS San Raffaele Hospital, Milan, Italy; 5 Gangarosa Department of Environmental Health, Rollins School of Public Health, Emory University, Atlanta, GA USA; 6 Department of Urology, Comprehensive Cancer Center, Medical University of Vienna, 1030 Vienna, Austria; 7 Department of Urology, La Croix du Sud Hospital, 31130 Quint-Fonsegrives, France; 8 Urology Division, Department of Surgical Sciences, University of Studies of Torino, 10124 Turin, Italy; 9 Department of Minimally Invasive and Robotic Urology, Wrocław Medical University, 50-367 Wrocław, Poland; 10 Department of Urology, Guys and St, Thomas’ NHS Foundation Trust, London, England; 11 Department of Obstetrics and Gynecology, Stanford University School of Medicine, Stanford, CA USA

**Keywords:** 5-alpha reductase inhibitor (5-ARi), Alpha blockers, Benign prostatic hyperplasia, Bladder cancer

## Abstract

**Background:**

Chemoprotective effect of 5-alpha reductase inhibitors (5-ARi) on bladder cancer (BCa) risk in men with Benign Prostatic Hyperplasia (BPH) has been explored with conflicting results. We sought to examine the effect of 5-ARi on new BCa diagnoses in a large US database.

**Methods:**

Men ≥ 50 y/o with a prescription for 5-ARi after BPH diagnosis were identified in the IBM® Marketscan® Research de-identified Databases between 2007 and 2016 and matched with paired controls. Incident BCa diagnoses were identified after BPH diagnosis and/or pharmacologic treatment. Multivariable regression modeling adjusting for relevant factors was implemented. Sub-group analyses by exposure risk were performed to explore the association between 5-ARi and BCa over time. Administration of alpha-blockers (α-B) w/o 5-ARi was also examined.

**Results:**

In total, *n* = 24,036 men on 5-ARi, *n* = 107,086 on 5-ARi plus alpha-blockers, and *n* = 894,275 without medical therapy for BPH were identified. The percentage of men diagnosed with BCa was 0.8% for the 5-ARi, 1.4% for the 5-ARi + α-B, and 0.6% for the untreated BPH group of incident BCa (adjusted hazard ratio [aHR], 0.90, 95% confidence interval [CI] 0.56 – 1.47), and 1.08, 95%CI 0.89 – 1.30, respectively). This was also true at both shorter (≤ 2 yr) and longer-term (> 2 yr) follow up. In addition, α-B alone had no change in BCa risk (HR 1.06, 0.86–1.30).

**Conclusions:**

We did not find any diminished risk of new BCa in men treated with 5-ARi (i.e., chemoprotective effect). The current report suggests that 5-ARi do not change a man’s bladder cancer risk.

**Supplementary Information:**

The online version contains supplementary material available at 10.1007/s00345-023-04551-4.

## Introduction

The American Cancer Society estimated 81,180 new cases (61,700 in men and 19,480 in women) in the United States (US) for 2022 and 17,100 deaths due to Bladder Cancer (BCa) [1]. Over 90% of BCa are transitional cell carcinoma histologically, with a mean age at diagnosis of 71 and 80% of cases occurring in those 65 and older [2]. Cigarette smoking is implicated in over a third of BCa patients diagnosed across the US and is a well-known risk factor for lowering rate of cancer-specific survival [3]. Apart rare (i.e., 5–8%) occupational exposures to aromatic amines, polycyclic aromatic or chlorinated hydrocarbons [4, 5], both the American and European Associations of Urology (AUA, EAU) Guidelines lack clear recommendations for other risk factors [6, 7] especially in comparison to other Genito-urinary (GU) malignancies such as prostate, kidney or testicular cancer (PCa, RCC, TCa). Androgen receptor (AR) signaling has been associated with urothelial cancer cell development and influencing survival outcomes prognosis both in animal models and humans [8]. However, the chemoprotective effect of androgen suppression therapy (AST) on incidence and survival related endpoints, such as recurrence, progression to invasive disease or death due to BCa has conflicting results. Specifically, the association between 5-alpha reductase inhibitors (5-ARi) and incident bladder cancer in men with Benign Prostatic Hyperplasia (BPH) is inconclusive and has only been demonstrated in single-institution series or limited population retrospective cohort analysis. The most up-to-date meta-analysis on this topic by Kim et al. [9] reported a significant protective effect on BCa incidence and mortality but studies were heterogenous. Given the widespread prevalence of BPH and its related AST administration in the US, we sought to examine the androgen modulation effect of 5-ARi on new BCa diagnoses in a large US sample.

## Materials and methods

### Data source

We performed a retrospective cohort analysis using administrative insurance claims data from IBM® MarketScan® Research Commercial and Medicare databases (https://doi.org/10.57761/ray7-1g16) containing individual-level, de-identified, inpatient, outpatient, outpatient pharmacy insurance billing claims, which enables longitudinal tracking of patients regardless of different sites of care and multiple treatment years, as well as inpatient and outpatient treatment, demographic data, diagnoses, procedures, and costs. International Classification of Disease Ninth and Tenth Revisions, Clinical Modification (ICD-9-CM, ICD-10-CM) codes, Current Procedural Terminology (CPT) codes were used to identify the study cohort, treatments, and comorbidities. This method has been used in other studies [10–12] and, given de-identified information, this study was exempt from informed consent requirements by Stanford University’s Institutional Review Board. Data for this project was accessed using the Stanford Center for Population Health Sciences Data Core. The PHS Data Core is supported by a National Institutes of Health National Center for Advancing Translational Science Clinical and Translational Science Award (UL1TR003142) and from Internal Stanford funding.

### Patients

Men at least 50 y/o were identified by ICD-9/10-CM and CPT diagnosis/treatment codes for BPH without concomitant active prescription of 5-ARi ± α-B between 2007 and 2016 to create the cohort of interest. Patients were enrolled in the database for at least 6 months before and 1 year after their initial BPH diagnosis. Medication codes were used to ensure 5-ARi and/or α-B were consistent for BPH treatment which included: Finasteride 5 mg; Finasteride 5 mg + Alfuzosin 10 mg; Dutasteride 0.5 mg, Dutasteride + Tamsulosin 0.4 mg; Alfuzosin 10 mg; Tamsulosin 0.4 mg; Silodosin 4 or 8 mg; Doxazosin 2 or 4 mg, Prazosin 0.5 or 1 mg; Terazosin 2 or 5 mg; Phenoxybenzamine 20 mg. The study population was then divided according to ‘BPH non-med users’ and ‘BPH med users’ sub-groups. Active medication users were further stratified on solely α-B or 5-ARi monotherapy and 5-ARi plus α-B combination. Given no pre-defined duration of protective effects of 5-ARi against BCa diagnosis is known, and with no evidence on the effect of interrupted use of 5ARi, we considered the first eligible period of usage per inclusion criterion. This approach was used to avoid assigning misleading exposure status for the risk of subsequent BCa diagnosis. Subjects with BPH diagnosis code and no evidence of 5-ARi or α-B use during the study time made up the control group.

Age at BPH diagnosis (index date), region, smoking status, prevalent comorbidities, and length of follow-up (i.e., exposure time) were considered. Baseline Charlson Comorbidity Index (CCI) was calculated according to Charlson and colleagues [13] and adapted according to Deyo and colleagues [14]. Patient insurance status was grouped as commercial or Medicare.

**Fig. 1:**
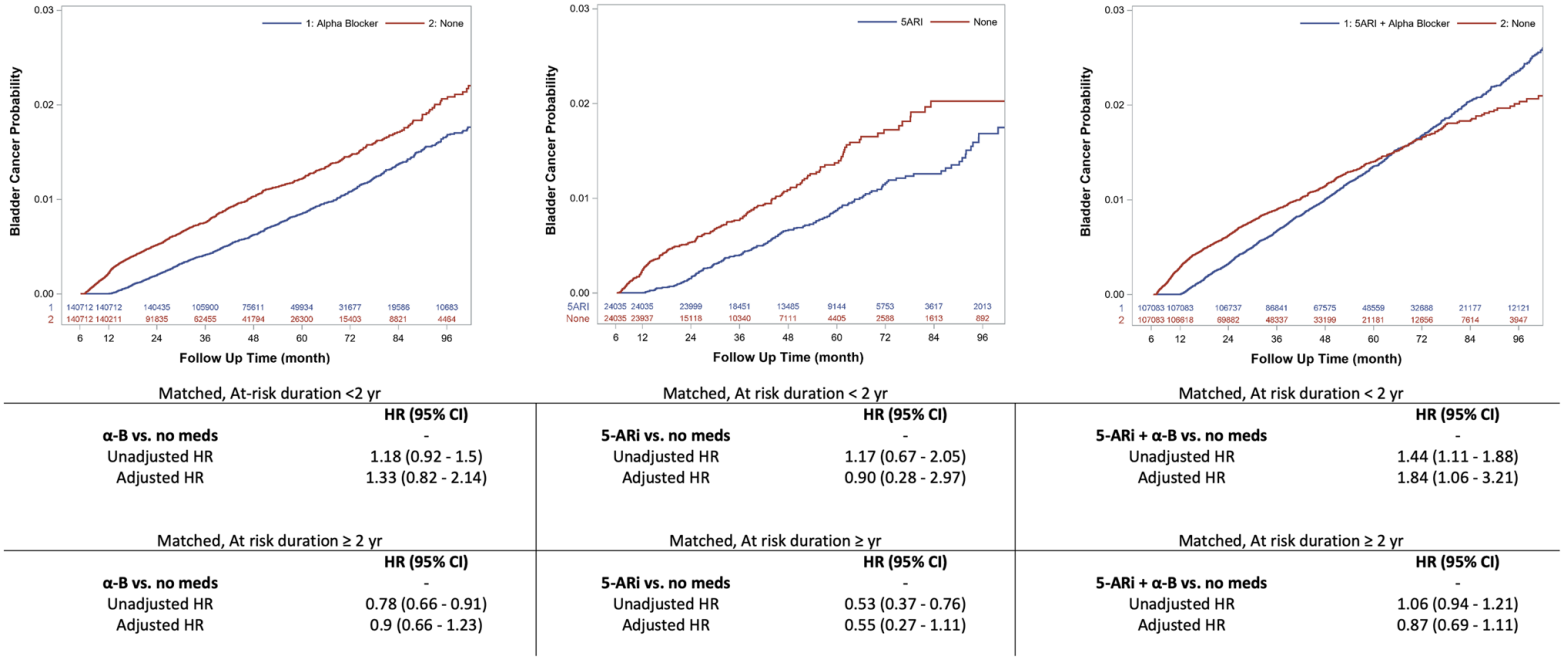
Univariable Kaplan-Meier curves and Multivariable Cox regression results in the PSM population for the risk for incident BCa in the different sub-groups of BPH-related medications stratified according to at-risk duration (i.e., <2 yr vs. ≥ 2 yr). *PSM* propensity score matching; *BCa* Bladder Cancer; *BPH* Benign Prostatic Hyperplasia; *yr* year

The incidence of primary BCa diagnosis by ICD-9/10 code was recorded following 1 yr after index date in all sub-groups. Patients with a BCa diagnosis before or within 6-months after the index date were removed. A history of current evidence of BCa at enrollment was an exclusion criterion hence all BCa diagnoses were considered new or incident cases. Patients who had undergone radical prostatectomy and/or androgen deprivation therapy (ADT) for the treatment of prostate cancer (PCa) were excluded. Pelvic radiation therapy was considered a time-dependent variable due to the possible effect on subsequent BCa development. A comprehensive list of codes and a flow chart diagram summarizing the analytical steps for data analysis and inclusion/exclusion criteria are in Supplementary Table 1 and Fig. 1, respectively.

### Outcome ascertainment

The primary aim was to ascertain the incidence of new BCa diagnosis in a large US cohort assuming AST (i.e., 5-ARi) in combination or not with α-B for BPH diagnosis. Additionally, we aimed to explore whether inhibiting the conversion of testosterone to DHT may lower the risk of later BCa incidence (i.e., chemoprotective effect). Patients were tracked over a pre-defined at-risk period 1-yr after the index date, allowing time for diagnosis of BCa and any residual effects of 5-ARi after discontinuation. Finally, we did not account for discontinued 5-ARi periods or patients who restarted 5-ARi after the initial period of use to avoid misleading exposure-related effects on BCa incidence.

### Statistical analysis

Patient demographics and clinical characteristics for those receiving 5-ARi w/o α-B vs. controls were reported by mean ± standard deviation (SD) or median and inter-quartile ranges (IQR) for continuous variables, frequencies and percentages for categorical variables. A propensity score matching (PSM) with a 1:1 ratio was implemented within each ‘BPH med users’ subgroup (i.e., 5-ARi or α-B alone and 5-ARi plus α-B) according to age at index date, CCI score, smoking status and/or concomitant diabetes. Kaplan–Meier curves were generated to explore the effect of 5-ARi and/or α-B on timing of subsequent BCa diagnosis.

A conditional multivariable Cox proportional hazards regression modeling was implemented to ascertain the effect of 5-ARi and/or α-B on the risk for later BCa diagnosis, adjusting for urologic-specific confounders such as urine cytology, history of hematuria, number of cystourethroscopy procedures, and pelvic radiation therapy considered as time dependent covariate. This was assessed for the whole cohort and repeated after re-matching the cohort by patients with shorter or longer-term follow-up time (i.e., < 2-yr vs. ≥ 2-yr) which is either considered a biological reliable risk exposure threshold as well as used in previous series to sub-analyze the same association. Analyses were two-sided with *p* < 0.05 considered significant, and performed using statistical software SAS, version 9.4 (SAS Institute Inc., Cary, NC, USA.).

## Results

### Study population

A total of *n* = 3,947,150 patients with ICD-9/10 associated BPH diagnosis were identified from 2007 to 2016 using MarketScan. Of these, prescription benefit was recorded in *n* = 3,028,614 cases. Approximately half of these (i.e., *n* = 1,382,144) had at least 6 months of enrollment time before and 1 year after BPH diagnosis and the majority were over 50 y/o (i.e., 1,184,517). *n* = 18,402 with BCa before or within 6 months after index date were excluded, reaching a final *n* = 1,166,115 patients in the cohort (Suppl. Fig. 1).

The vast majority of patients (*n* = 894,275, 76.7%) not associated with any BPH-related prescription defined the control group while the remaining *n* = 271,840 patients who met adequate time of exposure were associated with 5-ARi (*n* = 24,036, 8.8%), α-B (*n* = 140,718, 51.8%) or the combination of both (*n* = 107,086, 39.4%). Table 1 summarizes demographic, clinic and follow-up time on BCa incidence in the unmatched and matched population. The median enrollment time before the index date was similar across all four groups, ranging from a median of 1.6 yr (IQR: 0.9–3) to 2.1 yr (IQR: 1.1–3.9) with a median follow-up after the index date higher in exposed categories and slightly lower in control group (median 2.6 yr, IQR: 1.7–4.3).

**Table 1:** Baseline patient demographic, and clinical characteristics of the final cohort according to treatment modality (No meds vs. α-B vs. 5-ARi vs. 5-ARi + α-B) in the unmatched and propensity score matched population

Variables	Unmatched cohort				Matched cohorts					
	No medication	α-B	5-ARi	5-ARi + α-B	No medication	α-B	No medication	5-ARi	No medication	5-ARi + α-B
Sample size, *n*(%)	894.275	140.718	24.036	107.086	140.712	140.712	24.035	24.035	107.083	107.083
Age at index date, mean (SD)	62.3 (9.1)	65.7 (10)	67.4 (10.2)	68 (10.1)	65.7 (10.0)	65.7 (10.0)	67.4 (10.2)	67.4 (10.2)	68.0 (10.1)	68.0 (10.1)
*Age at index date, quartile distribution, n (%)*										
1st quartile	260,189 (29.09)	26,650 (18.94)	3407 (14.17)	14,027 (13.1)	26,650 (18.94)	26,650 (18.94)	3407 (14.18)	3407 (14.18)	14,027 (13.1)	14,027 (13.1)
2nd quartile	233,209 (26.08)	32,439 (23.05)	4913 (20.44)	20,647 (19.28)	32,439 (23.05)	32,439 (23.05)	4913 (20.44)	4913 (20.44)	20,647 (19.28)	20,647 (19.28)
3rd quartile	212,706 (23.79)	31,511 (22.39)	5615 (23.36)	23,581 (22.02)	31,511 (22.39)	31,511 (22.39)	5615 (23.36)	5615 (23.36)	23,581 (22.02)	23,581 (22.02)
4th quartile	188,171 (21.04)	50,118 (35.62)	10,101 (42.02)	48,831 (45.6)	50,112 (35.61)	50,112 (35.61)	10,100 (42.02)	10,100 (42.02)	48,828 (45.6)	48,828 (45.6)
Enrollment before index date (yr), median (IQR)	2.1 (1.1–3.9)	2.1 (1.1–3.7)	1.6 (0.9–3)	1.7 (0.9–3.1)	1.9 (0.9–3.6)	2.1 (1.1–3.7)	2.0 (1.1–3.8)	1.6 (0.9–3.0)	2.3 (1.1–4.3)	1.7 (0.9–3.1)
Follow-up after index date (yr), median (IQR)	2.6 (1.7—4.3)	4.2 (3–5.8)	4.3 (3.1–5.9)	4.8 (3.4–6.5)	2.4 (1.6–4.2)	4.2 (3.0–5.8)	2.6 (1.6–4.4)	4.3 (3.1–5.9)	2.7 (1.7–4.5)	4.8 (3.4–6.5)
*US Region, n (%)*										
Northeast	185,332 (20.72)	24,088 (17.12)	4727 (19.67)	18,633 (17.4)	30,712 (21.83)	24,086 (17.12)	5215 (21.7)	4727 (19.67)	21,059 (19.67)	18,633 (17.4)
North Central	225,267 (25.19)	40,497 (28.78)	6697 (27.86)	30,535 (28.51)	34,986 (24.86)	40,496 (28.78)	6067 (25.24)	6697 (27.86)	29,997 (28.01)	30,533 (28.51)
South	318,287 (35.59)	48,631 (34.56)	8319 (34.61)	36,593 (34.17)	49,185 (34.95)	48,630 (34.56)	8135 (33.85)	8319 (34.61)	36,474 (34.06)	36,593 (34.17)
West	156,020 (17.45)	26,588 (18.89)	4129 (17.18)	20,679 (19.31)	24,057 (17.1)	26,586 (18.89)	4435 (18.45)	4128 (17.17)	18,894 (17.64)	20,678 (19.31)
Unknown	9369 (1.05)	914 (0.65)	164 (0.68)	646 (0.6)	1772 (1.26)	914 (0.65)	183 (0.76)	164 (0.68)	659 (0.62)	646 (0.6)
CCI score, median (IQR) (range)	0 (0–2) (0–26)	1 (0–2) (0–19)	0 (0–2) (0–18)	1 (0–2) (0–20)	1 (0–2) (0–24)	1 (0–2) (0–19)	0 (0–2) (0–17)	0 (0–2) (0–18)	0 (0–2) (0–21)	1 (0–2) (0–20)
0	468,230 (52.36)	63,313 (44.99)	12,598 (52.41)	49,700 (46.41)	63,312 (44.99)	63,312 (44.99)	12,598 (52.42)	12,598 (52.42)	49,700 (46.41)	49,700 (46.41)
1	199,796 (22.34)	32,842 (23.34)	5334 (22.19)	24,774 (23.13)	32,840 (23.34)	32,840 (23.34)	5334 (22.19)	5334 (22.19)	24,771 (23.13)	24,771 (23.13)
2	90,287 (10.1)	16,534 (11.75)	2456 (10.22)	12,699 (11.86)	16,532 (11.75)	16,532 (11.75)	2456 (10.22)	2456 (10.22)	12,699 (11.86)	12,699 (11.86)
3 +	135,962 (15.2)	28,029 (19.92)	3648 (15.18)	19,913 (18.6)	28,028 (19.92)	28,028 (19.92)	3647 (15.17)	3647 (15.17)	19,913 (18.6)	19,913 (18.6)
*Comorbidities at index date, n (%)*										
Cancer	345,330 (38.62)	55,845 (39.69)	9672 (40.24)	42,489 (39.68)	55,708 (39.59)	55,844 (39.69)	9667 (40.22)	9671 (40.24)	45,297 (42.3)	42,488 (39.68)
Cardio-vascular disease	605,170 (67.67)	104,482 (74.25)	16,846 (70.09)	77,958 (72.8)	102,249 (72.67)	104,477 (74.25)	17,006 (70.76)	16,845 (70.09)	78,746 (73.54)	77,955 (72.8)
Hypertension	486,050 (54.35)	86,341 (61.36)	13,325 (55.44)	62,062 (57.96)	83,811 (59.56)	86,337 (61.36)	13,644 (56.77)	13,324 (55.44)	63,222 (59.04)	62,061 (57.96)
Diabetes	195,569 (21.87)	37,162 (26.41)	5034 (20.94)	26,365 (24.62)	37,158 (26.41)	37,158 (26.41)	5034 (20.94)	5034 (20.94)	26,364 (24.62)	26,364 (24.62)
Hyperlipidemia	511,366 (57.18)	78,565 (55.83)	12,882 (53.59)	54,897 (51.26)	84,222 (59.85)	78,561 (55.83)	13,213 (54.97)	12,882 (53.6)	60,338 (56.35)	54,896 (51.26)
Renal disease	53,200 (5.95)	11,784 (8.37)	1391 (5.79)	8148 (7.61)	11,211 (7.97)	11,784 (8.37)	1609 (6.69)	1390 (5.78)	8450 (7.89)	8148 (7.61)
Chronic pulmonary disease	134,396 (15.03)	25,243 (17.94)	3264 (13.58)	18,359 (17.14)	23,808 (16.92)	25,242 (17.94)	3421 (14.23)	3263 (13.58)	17,934 (16.75)	18,358 (17.14)
Liver disease	48,024 (5.37)	7130 (5.07)	828 (3.44)	4394 (4.1)	7603 (5.4)	7130 (5.07)	947 (3.94)	828 (3.44)	5110 (4.77)	4394 (4.1)
Depression	64,557 (7.22)	11,591 (8.24)	1225 (5.1)	6968 (6.51)	10,003 (7.11)	11,590 (8.24)	1503 (6.25)	1225 (5.1)	6939 (6.48)	6968 (6.51)
Peripheral vascular disease	78,247 (8.75)	16,105 (11.44)	2297 (9.56)	12,513 (11.69)	16,334 (11.61)	16,104 (11.44)	2416 (10.05)	2297 (9.56)	13,030 (12.17)	12,513 (11.69)
Cerebrovascular disease	86,161 (9.63)	18,552 (13.18)	2689 (11.19)	14,238 (13.3)	17,425 (12.38)	18,551 (13.18)	2843 (11.83)	2688 (11.18)	14,586 (13.62)	14,238 (13.3)
Heart disease	172,159 (19.25)	34,940 (24.83)	5544 (23.07)	27,268 (25.46)	33,488 (23.8)	34,940 (24.83)	5487 (22.83)	5543 (23.06)	27,136 (25.34)	27,266 (25.46)
Infectious diseases	197,684 (22.11)	33,021 (23.47)	4741 (19.72)	23,406 (21.86)	33,023 (23.47)	33,018 (23.46)	5372 (22.35)	4741 (19.73)	25,492 (23.81)	23,406 (21.86)
Smoking, n(%)	56,142 (6.28)	7656 (5.44)	780 (3.25)	4117 (3.84)	7651 (5.44)	7651 (5.44)	779 (3.24)	779 (3.24)	4114 (3.84)	4114 (3.84)
AST exposure (yr), median (IQR)	0.60 (0.09–1.13)	0 (0–0)	3.31 (2.33–4.82)	2.35 (0.85–4.11)	0.69 (0.14–1.17)	0 (0–0)	0.68 (0.13–1.14)	3.31 (2.33–4.82)	0.63 (0.10–1.15)	2.35 (0.85–4.11)
*AST exposure, n (%)*										
0	15,656 (21.35)	154 (95.65)	0 (0)	9485 (8.86)	2755 (19.78)	154 (95.65)	496 (20.1)	0 (0)	2342 (21.24)	9485 (8.86)
0–0.9	34,643 (47.24)	< 11	0 (0)	19,747 (18.44)	6370 (45.74)	< 11	1159 (46.96)	0 (0)	5191 (47.08)	19,747 (18.44)
1–1.9	22,730 (30.99)	0 (0)	3220 (13.4)	16,116 (15.05)	4735 (34)	0 (0)	799 (32.37)	3220 (13.4)	3434 (31.14)	16,115 (15.05)
2–2.9	124 (0.17)	0 (0)	6971 (29)	19,723 (18.42)	34 (0.24)	0 (0)	< 11	6970 (29)	20 (0.18)	19,721 (18.42)
3 +	187 (0.25)	< 11	13,845 (57.6)	42,015 (39.23)	32 (0.23)	< 11	< 11	13,845 (57.6)	39 (0.35)	42,015 (39.24)
Pelvic radiation therapy, *n* (%)	9174 (1.03)	1720 (1.22)	309 (1.29)	1546 (1.44)	1674 (1.19)	1720 (1.22)	269 (1.12)	309 (1.29)	1392 (1.30)	1546 (1.44)
BCa diagnosis during follow-up, *n* (%)	5516 (0.62)	1112 (0.79)	197 (0.82)	1464 (1.37)	1047 (0.74)	1112 (0.79)	213 (0.89)	197 (0.82)	1024 (0.96)	1464 (1.37)
BCa/1000-person-yr	0,16	0,14	0,15	0,23	0,20	0,14	0,23	0,15	0,24	0,23

Men without an active prescription for BPH were younger (mean, 62.3 ± 9.1 yr) and had reduced CCI scores at baseline (0, IQR: 0–2). They had a slightly higher percentage of active smokers in comparison with 5-ARi and 5-ARi plus α-B (6.3% vs. 3.3% and 3.8%, respectively). The most prevalent comorbidities at the index date were cardiovascular disease (67.7%), hyperlipidemia (57.2%), hypertension (54.4%), and any history of a prior cancer diagnosis excluding BCa (38.6%). However, no clinically relevant comorbidities of either overall male health or literature-based BCa risk factors were found to be imbalanced amongst the sub-groups.

Given the importance of the aforementioned factors on the risk for BCa, three separated sets of PSM were developed to mitigate the baseline sub-group impairments based on age, CCI score, smoking and diabetes. Results from PSM on the three different sub-groups are summarized in Table 1. Median at-risk time was 2.8 yr (IQR: 1.7–4.2 yr) for the whole cohort, yet patients in the 5-ARi plus α-B group presented with slightly higher exposure time than 5-ARi only (3.2 yr, IQR: 1.9–4.8 yr vs. 2.6 yr, IQR: 1.7–4.1). Among 5-ARi users, the relative percentages of prescription were 42.5% for dutasteride and 70.3% for finasteride while the most three utilized α-B were tamsulosin (76.4%), terazosin (14.9%), and doxazosin (14.4%). Out of these the one in combination with 5-ARi was tamsulosin (81.9%) and finasteride (68.8%).

### Outcomes

During follow-up, *n* = 8,289 patients or 0.7% of the sample developed BCa. However, at crude incidence assessment among sub-groups, the rate ranged between 0.74% and 0.96% in controls, and 0.82% and 1.37% in men exposed to AST. The rate of BCa diagnosis per 1000 person/yr, non-exposed men had the highest rate with 0.20, 0.23, and 0.24 respectively while lower rates were observed in cases of α-B or 5-ARi monotherapy (i.e., 0.14 and 0.15 respectively) and slightly increased in combination therapy (i.e., 0.23). Kaplan–Meier estimates of the matched population of both α-B or 5-ARi monotherapy exhibited a significantly lower failure rate (*p* < 0.001 and *p* < 0.001, respectively) while the combination of the two drugs did not result in additive protective effects. After adjustment for confounders associated with higher incidence for incidental BCa detection, the chemoprotective effect previously observed on univariable assessment vanished for α-B and 5-ARi monotherapy (adjusted Hazard Ratio [aHR]: 0.96, 95% Confidence Interval [CI] 0.74–1.24 and aHR: 1.14, 95%CI 0.64–2.02, respectively) as well as for 5-ARi plus α-B (aHR: 1.16, 95%CI 0.94–1.44). The associated risk was non-significant when the analysis was performed in re-matched population according to medication exposure time ≤ 2-yr vs. > 2-yr. Figure 1 displays a summary of Kaplan–Meier curves and risk estimates by both unadjusted and adjusted exposure time.

## Discussion

Our study represents the largest population-based cohort analysis in the US exploring the association of 5-ARi (monotherapy or in combination with α-B in men with BPH diagnosis) and incident BCa. Our findings do not support a chemoprotective effect of AST on BCa risk.

The physiological function of AR signaling in normal bladder remains unclear and few animal studies have demonstrated AR signaling contributing to the regulation of urine storage and urinary tract functions [15–17]. While studies in male rat-models suggest androgen deprivation results in the induction of bladder fibrosis and reduction of bladder capacity and compliance, androgen supplementation in castrated rats was found to augment urothelial thickness along with the quantity of smooth muscle fibers, and number of blood vessels in the lamina propria [18, 19]. The relationship of AR expression has also been examined in human urothelial cell lines and urothelial tumor tissue specimens demonstrating reduced AR signals in urothelial tumors compared with normal urothelial tissues [20, 21]. However, conflicting findings exist when examining AR protein promoters when stratifying for grade of disease (i.e., high vs. low) and stage of disease (i.e., non-muscle vs. muscle invasive bladder cancer [NMI, MIBC]) [20]. Sexual dimorphism of BCa incidence exists indirectly corroborating the influence of androgen modulation on urothelial cancer development. Chemoprotection is down-regulation from 5-ARi and consequent reduction in binding sites associated with tumor translocation genes and improvement of DNA double stranded break induced by AR signaling [22, 23]. Just recently, Abdel-Hafiz et al. conducted a study that examined the impact of Loss of the Y chromosome (LOY) on the prognosis of BCa (bladder cancer). The researchers found a consistent association between LOY and worse BCa prognosis. Specifically, in vitro experiments revealed similar growth rates between Y-positive (Y +) and Y-negative (Y-) tumors. However, in immune-competent hosts, Y- tumors exhibited greater aggressiveness compared to Y + tumors in a manner dependent on T cells. This suggests that BCa cells with LOY mutations affect T cell function, leading to T cell exhaustion and increased sensitivity to PD-1-targeted immunotherapy and thus potentially corroborating indirect role for androgens manipulation of BCa behavior [24].

Dekalo et al. [25] reported outcomes on *n* = 93,197 Canadian men on 5-ARi therapy (i.e., 52% dutasteride, and 48% finasteride) from the Canadian Ontario Drug Benefit database between 2003 and 2013. Similarly, patients were matched according to socio-demographic confounders and followed for the first eligible period of continuous 5-ARi use after BPH diagnosis. Exposure time was defined as 1-yr following drug initiation. Looking at risk for BCa diagnosis, there was no proven association in the shorter follow-up period in BCa diagnosis (HR 1.05, 95%CI 0.82 − 1.32) (i.e., < 2-yr follow-up). Similar trends were available for BCa mortality with no significant association in the adjusted model. There was a significant positive HR in the subset of longer follow-up for men on finasteride treatment. However, the work is limited to men over the age of 65, which may not apply to younger men.

Other important series on this topic have been led by secondary analysis of the Prostate, Lung, Colorectal, and Ovarian cancer (PLCO) screening trial and the Medical Therapy for Prostatic Symptoms (MTOPS) study. Morales et al. [26] evaluated data from a randomized US study focused on prostate-specific antigen and digital rectal examination screening on PCa mortality with a > 13-yr follow-up. Here, finasteride treatment was associated with decreased risk of BCa development (HR: 0.63, 95%CI 0.49–0.81). These findings are in contrast with the present work and the MTOPS study from Sathianathen et al. [27]. In the analysis of the MTOPS data, placebo, doxazosin, finasteride, or combination of both active treatments were not found to be protective for new BCa diagnosis. However, the self-reported usage of 5-ARi in the PLCO study compared to the prescription benefit report from our and MTOPS series is limited by a potential bias related to treatment ascertainment and use.

The present study is not devoid of limitations. As the dataset is administrative in nature and relies on accurate coding, there is a possibility of misclassification. Also, the retrospective design of the study, yet accounting on the largest sample size ever reported across the US on this topic, has per-definition intrinsic limitations allowing to rule out exploratory and limited associations. Additionally, drug prescription and diagnosis code including well known risk factors prevalence (e.g., smoking status), presented prior to access to insurance and entry into the database may not have been captured. Staging and information regarding characteristics of bladder tumors was unavailable. Moreover, survival data and patient ethnicity were not available, which is relevant given the evidence of the protective effect of 5-ARi in Caucasian/Hispanic groups, as opposed to African groups.

## Conclusion

In the US, the use of finasteride or dutasteride for BPH, whether used alone or in combination with α-B, was not associated with BCa incidence. This lack of reduction in risk was observed at both shorter and longer follow-up, indicating further testing is required before 5-ARi is advocated to lower BCa incidence. Our findings provide a robust evaluation of the association between 5-ARi use and BCa risk in the US. Future prospective studies are needed to confirm the findings, better understand the etiology of AR signaling in urothelial malignancies and consider if screening could be influenced for BPH men under AST.

### Supplementary Information

Below is the link to the electronic supplementary material.

Supplementary Figure 1. Flow chart depicting the decisional steps for creating the final cohort of interest by code matching (PNG 142 KB)

Supplementary Table 1. List of ICD-9/10 and CPT codes for Benign Prostatic Hyperplasia (BPH) and Bladder Cancer (BCa) diagnosis and treatments adopted to generate the cohort of interest (DOCX 160 KB)

## Data Availability

This is not applicable to the present study.
