# Supplementary figures and images for: 5-alpha reductase inhibitors (5-ARi) with or without alpha-blockers (α-B) for Benign Prostatic Hyperplasia do NOT lower the risk of incident Bladder Cancer: United States insurance claims data
Source: World J Urol. 2023 Aug 7;41(10):2783–91. doi: 10.1007/s00345-023-04551-4 (PMC10582125; doi:10.1007/s00345-023-04551-4)

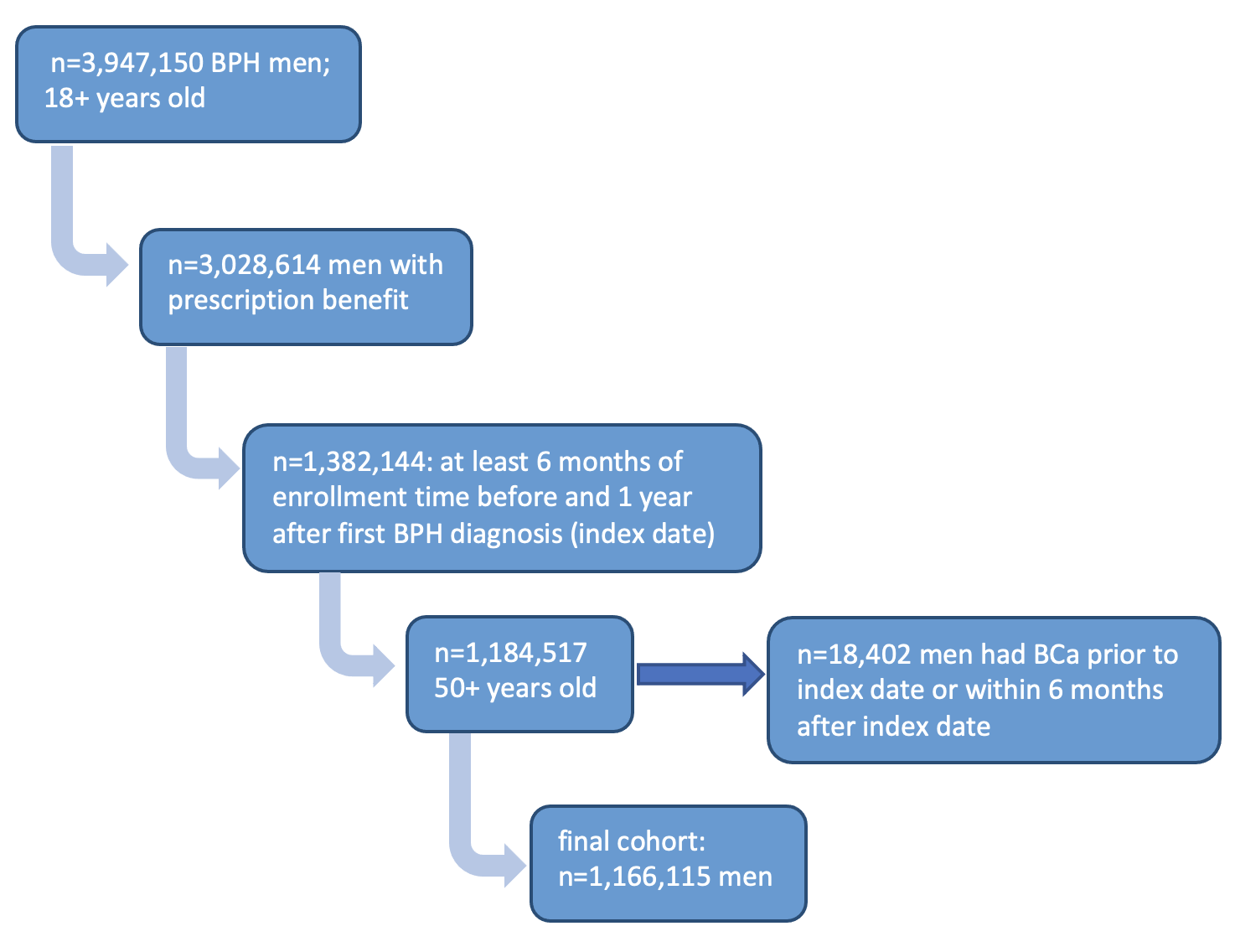

Supplement: Supplementary file 1 — Supplementary Figure 1. Flow chart depicting the decisional steps for creating the final cohort of interest by code matching (PNG 142 KB) [file 345_2023_4551_MOESM1_ESM.png]
