# Supplementary material for: 5-alpha reductase inhibitors (5-ARi) with or without alpha-blockers (α-B) for Benign Prostatic Hyperplasia do NOT lower the risk of incident Bladder Cancer: United States insurance claims data
Source: World J Urol. 2023 Aug 7;41(10):2783–91. doi: 10.1007/s00345-023-04551-4 (PMC10582125; doi:10.1007/s00345-023-04551-4)
Supplement: Supplementary file 2 [file 345_2023_4551_MOESM2_ESM.docx]

Benign localized hyperplasia of prostate (BPH)

ICD9/10 Code	Code Description
600	Hyperplasia of prostate
600.0	Hypertrophy (benign) of prostate
600.00	Hypertrophy (benign) of prostate without urinary obstruction and other lower urinary tract symptom (LUTS)
600.01	Hypertrophy (benign) of prostate with urinary obstruction and other lower urinary tract symptoms (LUTS)
600.1	Nodular prostate
600.10	Nodular prostate without urinary obstruction
600.11	Nodular prostate with urinary obstruction
600.2	Benign localized hyperplasia of prostate
600.20	Benign localized hyperplasia of prostate without urinary obstruction and other lower urinary tract symptoms (LUTS)
600.21	Benign localized hyperplasia of prostate with urinary obstruction and other lower urinary tract symptoms (LUTS)
600.3	Cyst of prostate
600.9	Hyperplasia of prostate unspecified
600.90	Hyperplasia of prostate, unspecified, without urinary obstruction and other lower urinary symptoms (LUTS)
600.91	Hyperplasia of prostate, unspecified, with urinary obstruction and other lower urinary symptoms (LUTS)
D29.1	Benign neoplasm of prostate
N40.2	Nodular prostate without lower urinary tract symptoms
N40.3	Nodular prostate with lower urinary tract symptoms
N42.83	Cyst of prostate

Radiation therapy

ICD-10-PCS		ICD-9-PCS
DV000ZZ	Beam Radiation of Prostate using Photons <1 MeV	92.22, or 92.24
DV001ZZ	Beam Radiation of Prostate using Photons 1 - 10 MeV	92,24
DV002ZZ	Beam Radiation of Prostate using Photons >10 MeV	92,24
DV003ZZ	Beam Radiation of Prostate using Electrons	92,25
DV004ZZ	Beam Radiation of Prostate using Heavy Particles (Protons,Ions)	92,26
DV005ZZ	Beam Radiation of Prostate using Neutrons	92,26
DV006ZZ	Beam Radiation of Prostate using Neutron Capture	92,26
DV1097Z	High Dose Rate (HDR) Brachytherapy of Prostate using Cesium 137 (Cs-137)	92,23
DV1098Z	High Dose Rate (HDR) Brachytherapy of Prostate using Iridium 192 (Ir-192)	92,23
DV1099Z	High Dose Rate (HDR) Brachytherapy of Prostate using Iodine 125 (I-125)	92,23
DV109BZ	High Dose Rate (HDR) Brachytherapy of Prostate using Palladium 103 (Pd-103)	92,23
DV109CZ	High Dose Rate (HDR) Brachytherapy of Prostate using Californium 252 (Cf-252)	92,23
DV109YZ	High Dose Rate (HDR) Brachytherapy of Prostate using Other Isotope	92,23
DV10B6Z	Low Dose Rate (LDR) Brachytherapy of Prostate using Cesium 131 (Cs-131)	None
DV10B7Z	Low Dose Rate (LDR) Brachytherapy of Prostate using Cesium 137 (Cs-137)	92,23
DV10B8Z	Low Dose Rate (LDR) Brachytherapy of Prostate using Iridium 192 (Ir-192)	92,23
DV10B9Z	Low Dose Rate (LDR) Brachytherapy of Prostate using Iodine 125 (I-125)	92,23
DV10BB1	Low Dose Rate (LDR) Brachytherapy of Prostate using Palladium 103 (Pd-103), Unidirectional Source	None
DV10BCZ	Low Dose Rate (LDR) Brachytherapy of Prostate using Californium 252 (Cf-252)	92,23
DV10BYZ	Low Dose Rate (LDR) Brachytherapy of Prostate using Other Isotope	92,23
DV20DZZ	Stereotactic Other Photon Radiosurgery of Prostate	92.31 or 92.39
DV20HZZ	Stereotactic Particulate Radiosurgery of Prostate	92,33
DV20JZZ	Stereotactic Gamma Beam Radiosurgery of Prostate	92,32
DVY07ZZ	Contact Radiation of Prostate	92,21
DVY08ZZ	Hyperthermia of Prostate	99,85
DVY0CZZ	Intraoperative Radiation Therapy (IORT) of Prostate	92,29
DVY0FZZ	Plaque Radiation of Prostate	92,29
DVY0KZZ	Laser Interstitial Thermal Therapy of Prostate	17,69

Malignant neoplasm of bladder (BCa)

ICD9/10 Code	Code Description
C67.0	Malignant neoplasm of trigone of bladder
C67.1	Malignant neoplasm of dome of bladder
C67.2	Malignant neoplasm of lateral wall of bladder
C67.3	Malignant neoplasm of anterior wall of bladder
C67.4	Malignant neoplasm of posterior wall of bladder
C67.5	Malignant neoplasm of bladder neck
C67.6	Malignant neoplasm of ureteric orifice
C67.7	Malignant neoplasm of urachus
C67.8	Malignant neoplasm of overlapping sites of bladder
C67.9	Malignant neoplasm of bladder, unspecified
188.0	Malignant neoplasm of trigone of urinary bladder
188.1	Malignant neoplasm of dome of urinary bladder
188.2	Malignant neoplasm of lateral wall of urinary bladder
188.3	Malignant neoplasm of anterior wall of urinary bladder
188.4	Malignant neoplasm of posterior wall of urinary bladder
188.5	Malignant neoplasm of bladder neck
188.6	Malignant neoplasm of ureteric orifice
188.7	Malignant neoplasm of urachu
188.8	Malignant neoplasm of other specified sites of bladder
188.9	Malignant neoplasm of bladder, part unspecified

Comorbidities	ICD-9		ICD-10	
cancer, without Pca or Bca	140-209	140-239	C00 - C96	C00-D49
cardio-vascular disease	390-459	390-459	I00 - I99	I00-I99
hypertension	401-405	401-405	I10-I16	I10-I16
diabetes	250	250	E08-E13	E08 - E13
hyperlipidemia	272.0-272.4	272.0-272.4	E78.0, E78.1, E78.2, E78.4, E78.5	E78
renal disease	403.01, 403.11, 403.91, 404.02, 404.03, 404.12, 404.13, 404.92, 404.93, 580 - 588, V42.0, V45.1	580-588	N17, N18, N19	N00-N19
chronic pulmonary disease	490 - 496	490-496	J40 - J47	J40-47
liver disease	070.22, 070.23, 070.32, 070.33, 070.44, 070.54, 070.6, 070.9, V42.7	570-573	K70 - K77, B18, B19	K70-77
depression	311, 296.2, 296.3, 298.0, 300.4, 309.1	296.2, 296.3, 298.0, 300.4, 309.1, 311	F32, F33	F32, F33
peripheral vascular disease	440 - 443	440-443	I70 - I79	I70-I79
cerebrovascular disease	430-438	430-438	I60-I69	I60 - I69
heart disease	410-414	410-414, 420-429	I20-I25	I20-I52
infectious diseases	001-139	001-136	A00-B99	A00-B99
smoking	305.1, V15.82		F17.200, Z87.891	

BCa Therapeutic codes

ICD-9 codes	Description	ICD-10 codes	Description	CPT code	Description
57.31	Cystoscopy through artificial stoma convert	0TJB8ZZ	Inspection of Bladder, Via Natural or Artificial Opening Endoscopic	52234	Cystourethroscopy with fulguration (including cryosurgery or laser surgery) and or resection of SMALL bladder tumor(s) (0.5 up to 2.0 cm).
57.32	Other cystoscopy	0TJB8ZZ	Inspection of Bladder, Via Natural or Artificial Opening Endoscopic	52235	Cystourethroscopy with fulguration (including cryosurgery or laser surgery) and or resection of MEDIUM bladder tumor(s) (2.0 to 5.0 cm).
57.33	Closed [transurethral] biopsy of bladder	0TBB3ZX	Excision of Bladder, Percutaneous Approach, Diagnostic	52240	Cystourethroscopy with fulguration (including cryosurgery or laser surgery) and or resection of LARGE bladder tumor(s).
57.34	Open biopsy of bladder	0TBB4ZX	Excision of Bladder, Percutaneous Endoscopic Approach, Diagnostic	52224	Trans-uretrhal fulguration of bladder tumor
57.39	Other diagnostic procedures on bladder	0TBB7ZX	Excision of Bladder, Via Natural or Artificial Opening, Diagnostic	51530	Trans-uretrhal resection of bladder tumor
		0TBB8ZX	Excision of Bladder, Via Natural or Artificial Opening Endoscopic, Diagnostic	51020 - 51030	fulguration or cryosurgical destruction of bladder lesions through an incision
57.49	Other transurethral excision or destruction of lesion or tissue of bladder	0TBB0ZX	Excision of Bladder, Open Approach, Diagnostic	52204	Cystourethroscopy, with biopsy(s)
57.59	Open excision or destruction of other lesion or tissue of bladder	0T5B7ZZ	Destruction of Bladder, Via Natural or Artificial Opening	52000	
		0T5B8ZZ	Destruction of Bladder, Via Natural or Artificial Opening Endoscopic		
57.6	Partial cystectomy	0TBB7ZZ	Excision of Bladder, Via Natural or Artificial Opening		
57.71	Radical cystectomy	0TBB8ZZ	Excision of Bladder, Via Natural or Artificial Opening Endoscopic		
57.79	Other total cystectomy	0T5B0ZZ	Destruction of Bladder, Open Approach		
		0T5B3ZZ	Destruction of Bladder, Percutaneous Approach		
CPT		0T5B4ZZ	Destruction of Bladder, Percutaneous Endoscopic Approach		
51550-51565	partial cystectomy	0T5C0ZZ	Destruction of Bladder Neck, Open Approach		
